# Pleomorphic Adenoma of the Soft Palate: Case Report

**DOI:** 10.1155/carm/8048933

**Published:** 2025-08-06

**Authors:** Yuliya Menchisheva, Saule Mussabekova, Dana Menzhanova, Shynggys Duisenbay, Magomed Khairoyev, Kamil Zubanov

**Affiliations:** ^1^Department of Life Course & Population Sciences, King's College London, Franklin Wilkins Building, 150 Stamford Street, London SE1 9NH, UK; ^2^Department of Morphology, School of Medicine, Karaganda Medical University, Karaganda, Kazakhstan; ^3^Department of Oral and Maxillofacial Surgery, S.D. Asfendiyarov Kazakh National Medical University, 94 Tole Bi Street, Almaty 050000, Kazakhstan

## Abstract

**Background:** Pleomorphic adenoma is the most prevalent benign tumor of the salivary glands. While it primarily affects the parotid gland, it can also arise from minor salivary glands in the soft palate.

**Presentation:** A case of a 28-year-old female who presented with a painless, slowly enlarging mass on the soft palate was reported. CT revealed a well-circumscribed lesion measuring 2.5 × 2.0 cm. Complete surgical excision was performed, and histopathological analysis confirmed the diagnosis of pleomorphic adenoma.

**Conclusion:** Pleomorphic adenoma of the palate is an important entity to consider in the differential diagnosis of salivary gland neoplasms. Early recognition and complete surgical removal are key to preventing recurrence and malignant transformation.

## 1. Introduction

Pleomorphic adenoma (PA) is a benign tumor that represents around 60% of all salivary gland neoplasms [1]. While the parotid gland is most frequently involved, PA of the soft palate arising from minor salivary glands is uncommon clinical occurrence, representing a small proportion (10%–25%) of all salivary gland neoplasms [2]. While PAs of the minor salivary glands most frequently occur in the palate, these tumors can also develop in locations such as the upper lip, floor of the mouth, cheek, larynx, and trachea [3]. Malignancy develops in roughly 50% of minor salivary gland tumors, which is a clinically important difference, as this rate is notably greater than the rate seen in major salivary gland tumors [4, 5].

PA is the most frequently observed benign tumor, while adenoid cystic carcinoma (ACC) is a common malignant tumor [6, 7]. Accurate differentiation between PA and ACC is crucial for surgical planning, as treatment varies from simple excision with clear margins to radical resection involving adjacent tissues, based on tumor type [4].

PA of the soft palate usually appears as a gradually enlarging, painless mass that can remain asymptomatic for long periods [8]. The preferred treatment is surgical removal with sufficient margins due to the risk of recurrence and potential for malignant transformation in long-standing cases [1, 9]. This case report highlights a rare presentation of PA in the soft palate of a young adult female.

## 2. Case

A 28-year-old female presenting with a painless soft palate mass was referred to the Department of Oral and Maxillofacial Surgery at Hospital 5 in Almaty, Kazakhstan. The patient reported a painless swelling in the soft palate, which had been present for about 2 months. The patient denied any difficulty in swallowing, speech, or breathing. No history of trauma, systemic illness, or tobacco consumption was indicated. From the patient's perspective, the primary concern was the visible swelling on the soft palate and its potential impact on appearance and speech, which motivated decision to undergo prompt surgical removal.

**Figure 1:**
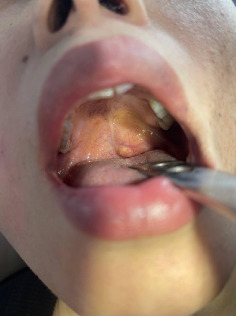
Preoperative view of the lesion demonstrated swelling of the left soft palate.

Intraoral examination revealed a well-defined, firm, nontender, dome-shaped, mobile mass on the left side of the soft palate, covered by normal mucosa. The lesion measured approximately 2.0 × 2.0 cm. There was no ulceration or bleeding.  Figure 1 shows the preoperative dome-shaped swelling on the left soft palate.

Fine-needle aspiration cytology showed that the biopsy sample contained spindle-shaped or plasmacytoid epithelioid myoepithelial cells within a chondromyxoid stroma.

**Figure 2:**
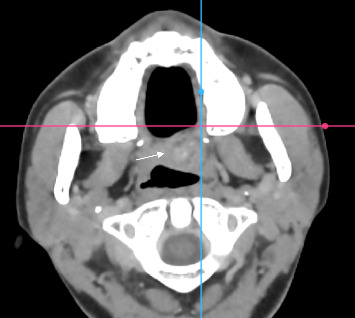
A computed tomography scan of the well-defined soft-tissue mass measuring 2.5 × 2.0 cm, clearly separated from the surrounding structures.

A computed tomography (CT) scan was performed to evaluate the extent and characteristics of the palatal lesion. The imaging revealed a well-defined, oval-shaped soft tissue mass located on the left side of the soft palate, measuring approximately 2.5 × 2.0 cm in its greatest dimensions. The margins of the lesion were smooth and well-circumscribed, with no evidence of infiltration into the adjacent musculature or osseous structures. The capsule appeared intact, suggesting a benign, encapsulated process.  Figure 2 displays the CT scan where the well-defined lesion appears separated from surrounding structures. The CT scan showed no signs of palatal bone erosion or cortical involvement.

While the CT scan revealed a well-defined mass, there was no clear radiographic evidence of encapsulation, which is not uncommon in PA of the soft palate due to resolution limitations.

**Figure 3:**
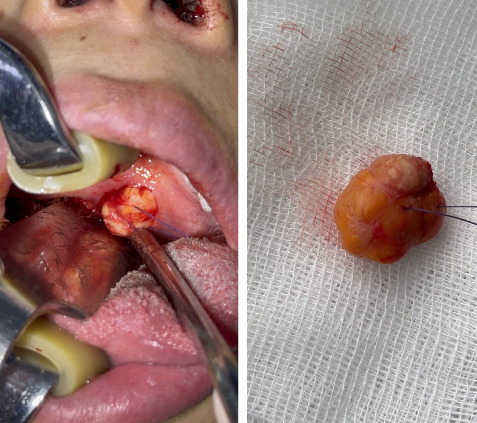
Intraoperative image of the mass being dissected from the underlying tissues.

The mass was surgically removed under general anesthesia using a transoral approach. General anesthesia was preferred due to patient anxiety and location near the oropharynx.  Figure 3 demonstrates intraoperative dissection of the mass. Endotracheal intubation was performed, although not visible in intraoperative photo due to angle. Although the lesion was small, excision included a rim of healthy tissue. The tumor was completely removed. No capsular breach was noted. The postoperative course was uneventful.

**Figure 4:**
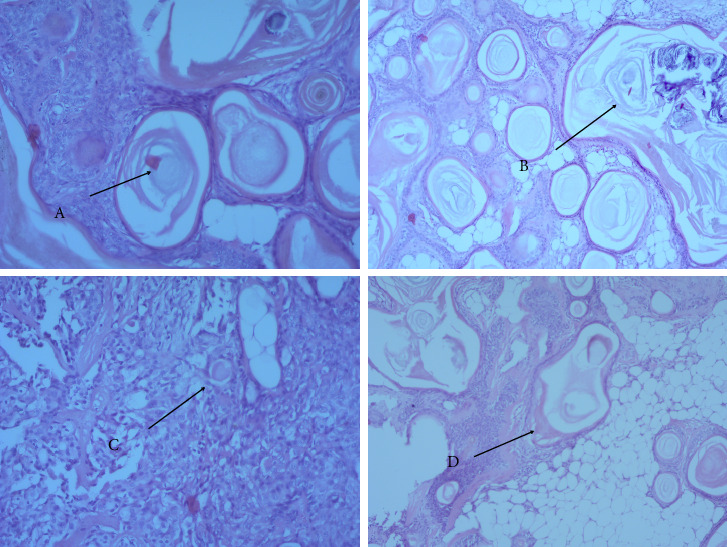
Histopathology examination: multiple horny cysts (A), areas of cyst calcification (B), layers of polymorphous epidermoid cells (C), and basal cell islands (D) (hematoxylin-eosin stain, original magnification ×400).

Histopathological analysis verified the diagnosis of PA, revealing a combination of epithelial and myoepithelial components within a chondromyxoid stroma. The tumor exhibited significant architectural complexity, including numerous rounded keratinous cysts composed of keratotic-cystic structures containing horny masses—a finding that, while rare, is characteristic of certain PA subtypes.  Figure 4(A) depicts these horny cysts in detail. In several regions, calcification of the horny masses was observed, represented by crumbling calcified deposits within the cystic spaces (Figure 4(B)). These changes likely reflect chronicity and degenerative processes within the tumor. A giant cell reaction was noted in areas where the cyst wall had ruptured, suggesting a secondary inflammatory response to epithelial disruption. The intercystic areas were populated by layers of polymorphous epidermoid cells, which exhibited variability in nuclear and cytoplastic features, consistent with the tumor's diverse cellular makeup (Figure 4(C)). In addition, basal cell islands were present within the stroma (Figure 4(D)), appearing as small, well-circumscribed nests, further supporting the benign epithelial origin of the lesion. No evidence of vascular invasion, necrosis, or cellular atypia was found, and the tumor lacked signs of malignant transformation. Overall, Figure 4 illustrates these key histological findings—horny cysts (A), cystic calcification (B), polymorphous epidermoid layers (C), and basal cell nests (D)—which together reinforce the diagnosis of PA and highlight the unique histopathological features of this case.

Although immunohistochemical staining was not performed, the diagnosis was confirmed based on well-established classical morphological features. However, the absence of IHC may be considered a limitation in excluding rare variants or malignancy.

The patient was reviewed at 6 months postoperatively, with no clinical evidence of recurrence or complications. The surgical site showed satisfactory healing. A follow-up examination with imaging and intraoral photographs is scheduled for the 1-year postoperative mark to further document long-term healing.  Table 1 illustrates clinical timeline of the patient's case.

## 3. Discussion

PA, the most frequently occurring salivary gland neoplasm, comprises approximately 60% of all salivary gland tumors [10]. PA of the minor salivary glands is relatively rare (10%), but when it occurs, the palate is the most frequent intraoral location due to the abundance of minor salivary glands in that region [3]. PA can also occur in unusual locations such as the lip, sinuses, larynx, epiglottis, and trachea [11, 12]. Heterotopic PAs, occurring outside typical salivary gland locations, have been reported in the lacrimal gland, maxilla, auricle, tongue, and throughout the alimentary and respiratory systems [13–17].

PAs most commonly affect patients in the fourth and sixth decades of life, with a female predominance [3]. The sex of the case in the current study aligns with that reported in most previous studies, while the age differs from what has typically been described [1, 3].

These tumors typically present as painless, slow-growing masses and are often discovered incidentally [18, 19]. Clinically, PAs originating in the hard and soft palate often present as nonulcerated, noninflamed, firm or rubbery submucosal masses [20–22]. Our findings are consistent with previously reported soft palate PA cases, such as those by Al Sahman et al., Liu et al., Ndiaye et al., Bovino et al., Daryani et al., Daniels et al., which similarly describe slow-growing, painless submucosal lesions in young to middle-age adults.

The differential diagnosis for palatal swelling includes benign salivary gland tumors, ACC, mucoepidermoid carcinoma, mucoceles, and benign connective tissue tumors [1, 23]. Imaging plays a crucial role in defining the extent of the lesion and its relationship to adjacent structures. CT and MRI are utilized in the preoperative assessment and treatment planning of tumors of the soft palate [24]. CT provides excellent bony detail and was useful in current case to rule out palatal bone invasion. MRI, however, offers superior soft tissue contrast and may be preferred for surgical planning in cases with suspected perineural spread or deep tissue involvement. Fine-needle aspiration biopsy (FNAB) is also used in combination with radiologic evaluation. However, the results of FNAB may have low sensitivity (reported between 60% and 80%) and may yield inconclusive results due to the tumor's mixed cellularity. Definitive diagnosis depends on histopathological evaluation [25].

Salivary gland tumors exhibit significant morphological diversity, making accurate diagnosis in pathology challenging [26]. Histologically, PAs are characterized by a combination of epithelial and myoepithelial cells embedded in a variable stromal background [1]. In 1976, Seifert et al. presented classification of PA, categorising the tumors into four types: Type I was primarily composed of the myxoid variant; Type II included a mixture of myxoid and cellular components; Type III was mainly cellular; and Type IV exhibited a highly cellular composition [27]. According to the 2022 WHO classification of salivary gland tumors, PAs can be subtyped based on the relative dominance of stromal and cellular elements: myxoid (> 80% stroma), classic or mixed (30%–80%), and cellular (< 30%) [28]. As observed in the current case study, the tumor displayed a balanced composition of epithelial and myoepithelial cells embedded in a moderately prominent chondromyxoid stroma, accompanied by keratinous cysts and focal calcifications. These features are characteristic of the classic (mixed) subtype.

This case is distinguished by the patient's relatively young age of 28, which is less common than the typical presentation. Additionally, histopathological examination revealed unusual features such as calcified horny masses and giant cell reaction, which are rarely described in the literature, enhancing the case's novelty.

Table 2 outlines the key clinical, imaging, and histopathological features that help distinguish PA from other palatal masses, including ACC, and benign mesenchymal tumors.

The encapsulated nature of the tumor allows for surgical excision with a good prognosis. However, incomplete removal or capsule rupture may lead to recurrence [3]. In this case, the tumor was excised with surrounding soft tissue, but not down to the periosteum. While appropriate for small, well-circumscribed benign lesions, excision extending to the palatal bone may further reduce the risk of recurrence and is recommended in larger or recurrent cases [29].

Malignant transformation, although uncommon, is a serious complication and underscores the importance of early and complete excision [30]. Although no signs of recurrence were observed during the 6-month follow-up period, this duration is relatively short given the known potential for PAs to recur even several years after initial treatment [31]. Therefore, long-term follow-up, including periodic clinical examination and imaging when indicated, is strongly recommended for at least 5 years to ensure early detection of any recurrence or rare malignant transformation [32].

PA is a benign tumor, however malignant transformation can occur, most commonly into carcinoma ex PA (Ca ex PA) [33]. This transformation is a rare but serious complication, with reported rates ranging from 1.5% to 22.2%, depending on tumor duration, location, and completeness of prior excision [32, 34]. Several risk factors have been identified that increase the likelihood of malignant change: long-standing tumors, particularly those present for more than 10 years; inadequate or incomplete surgical excision, especially with capsular rupture or tumor spillage; multiple recurrences of previously excised PAs; larger tumor size (more than 2 cm); older patient age (over 40 years), long years of smoking history (more than 10 years) [35–37]. Histologically, Ca ex PA often shows areas of high-grade epithelial malignancy arising within or adjacent to residual benign PA tissue [37]. Given these risks, it is critical to perform complete surgical excision with intact margins during the initial surgery and to maintain long-term clinical follow-up to monitor for recurrence or malignant progression.

## 4. Conclusion

PA of the palate should be regarded as an important differential diagnosis for masses in the palatal region. A thorough clinical and radiographic evaluation followed by complete surgical excision ensures excellent prognosis. Early diagnosis and appropriate management are essential to prevent recurrence and malignant transformation.

## Figures and Tables

**Table 1:** Clinical timeline of the patient's case.

Week 0	Onset of painless palatal swelling
Week 8	Clinical evaluation and referral
Week 9	CT imaging and FNAC performed
Week 10	Surgical excision under general anesthesia
Week 12	Histopathological diagnosis confirmed
Month 6	Follow-up: No recurrence
Month 12	Scheduled a follow-up clinical examination, imaging and photographic documentation

**Table 2:** Differential diagnosis of pleomorphic adenoma of the palate.

Feature	Pleomorphic adenoma (PA)	Adenoid cystic carcinoma (ACC)	Mucoepidermoid carcinoma (MEC)	Benign connective tissue tumor
Clinical presentation	Painless, slow-growing, dome-shaped submucosal mass, firm and mobile	Frequently painful, fixed mass, often associated with perineural spread invasion	May be painless or tender, often fluctuating in size	Well-defined, painless, slow-growing mass
Typical age group	30–60 years	40–60 years	20–60 years	Any age
Common site (palate)	Hard or soft palate (especially posterior lateral region)	Hard palate	Palate, buccal mucosa, retromolar area	Any oral mucosal surface
Imaging (CT/MRI)	Well-circumscribed, noninvasive, soft tissue mass, usually no bone involvement	Poorly defined margins, infiltrative growth, possible bone erosion	Mixed solid-cystic lesion, variable enhancement	Homogeneous, well-circumscribed, no bone involvement
Capsule	Often encapsulated	Nonencapsulated, infiltrative	May be encapsulated or show cystic spaces	Encapsulated or sharply demarcated
Histopathology	Epithelial and myoepithelial cells in chondromyxoid stroma, calcifications may be present	Cribriform or tubular, solid patterns (“Swiss cheese”), perineural invasion common	Mucous, intermediate, and epidermoid cells, cystic architecture	Dense fibrous connective tissue, no epithelial elements
FNAC findings	Plasmacytoid myoepithelial cells, chondromyxoid background	Basaloid cells in cribriform structures, difficult to distinguish from PA	Mucin-containing cells, variable yield	Spindle cells, low cellularity
Recurrence risk	Low with complete excision, increases with capsular rupture	High, even with wide excision due to perineural spread	Intermediate recurrence risk	Rare
Malignant potential	Rare, but possible with long-standing or recurrent tumors	Malignant	Low to intermediate grade malignancy	Benign, no transformation

## Data Availability

The data that support the findings of this study are available from the corresponding author upon reasonable request.
